# Hemispherical Solar Distiller Performance Utilizing Hybrid Storage Media, Paraffin Wax with Nanoparticles: An Experimental Study

**DOI:** 10.3390/molecules27248988

**Published:** 2022-12-16

**Authors:** Ravishankar Sathyamurthy, Wael M. El-Maghlany, Mohammed El Hadi Attia, A. E. Kabeel, Mohamed Abdelgaied, Moataz M. Abdel-Aziz, A. S. Abdullah, S. Vasanthaseelan

**Affiliations:** 1 Department of Mechanical Engineering, King Fahd University for Petroleum and Minerals, Dammam 31261, Saudi Arabia; 2 Interdisciplinary Research Center for Renewable Energy and Power Systems (IRC-REPS), King Fahd University of Petroleum and Minerals, Dhahran 31261, Saudi Arabia; 3 Mechanical Engineering Department, Faculty of Engineering, Alexandria University, Alexandria 5424041, Egypt; 4 Department of Physics, Faculty of Science, University of El Oued, El Oued 39000, Algeria; 5 Mechanical Power Engineering Department, Faculty of Engineering, Tanta University, Tanta 31733, Egypt; 6 Faculty of Engineering, Delta University for Science and Technology, Gamasa 7730103, Egypt; 7 Mechanical Power Engineering Department, Faculty of Engineering, Horus University, New Damietta 34517, Egypt; 8 Mechanical Engineering Department, College of Engineering, Prince Sattam bin Abdulaziz University, Wadi Addawaser 11991, Saudi Arabia; 9 Faculty of Engineering, Tanta University, Tanta 31733, Egypt; 10 Department of Mechanical Engineering, KPR Institute of Engineering and Technology, Arasur, Coimbatore 641407, Tamil Nadu, India

**Keywords:** desalination, hemispherical solar distiller, PCM, Al_2_O_3_ nanoparticles, thermal conductivity, efficiency, fresh water

## Abstract

The traditional method of obtaining fresh water for drinking is by burning fossil fuels, emitting greenhouse gases into the atmosphere. However, renewable energy is gaining more traction since it is available free of cost for producing fresh water. In this study, Al_2_O_3_ nanoparticles were distributed in a phase change material (paraffin wax) that had been fixed at a hemispherical distiller water basin. Three scenarios with three hemispherical distillers were examined. A conventional hemispherical distiller (CHD), a conventional hemispherical distiller with paraffin wax as a phase change material (CHD-PCM), and a conventional hemispherical distiller with PCM partially filled with Al_2_O_3_ nanoparticles (CHD-N-PCM) were tested under the same climatic conditions. The experimental results showed that CHD gave a daily yield of 4.85 L/m^2^/day, while CHD-PCM increased the yield to up to 6.2 L/m^2^/day with a 27.84% daily yield enhancement. The addition of Al_2_O_3_ nanoparticles to paraffin wax CHD-N-PCM improved hemispherical distillate yield up to 8.3 L/m^2^/day with a 71.13% increase over CHD yield.

## 1. Introduction

Many challenges and problems have recently afflicted the Earth, making it difficult to live on it. One of these issues is global warming, which is caused by increased carbon dioxide diffusion rates in the atmosphere as a result of fossil fuels [1,2,3,4,5]. Increased public awareness of the environmental risks posed by global warming, as well as fluctuating oil prices and dwindling global fossil fuel reserves, have prompted governments to turn to alternative energy sources to meet their energy needs in a clean, efficient, renewable, and long-term manner [6]. Providing water suitable for human, industrial, and agricultural consumption is one of the global challenges that must be addressed in order to achieve the long-term aspirations that are threatened by the problem of water scarcity [7,8,9,10]. The effect of the phase change material in the solar distillation unit for heat recovery to improve thermal performance was experimentally studied by Al-harahsheh et al. [11]. According to the results, the PCM improved the CSS performance by 40%. With high density and thermal conductivity, copper oxide nanoparticles were doped in paraffin wax and used as energy storage for augmented fresh water yield by Abdullah et al. [12]. The authors found that using paraffin wax via CuO nanoparticles achieved a 108% yield improvement compared to the reference still. The effect of mixing the PCM with CuO nanoparticles on the corrugated tray in the absorber of solar still was experimentally studied by Abdullah et al. [13]. The authors concluded that the corrugated solar still with CuO nanoparticles produced a 122% yield gain over the conventional still. Using coconut oil-based PCM and CuO nanoparticles, Al-Jethelah et al. [14] experimentally studied the melting point of PCM in an opened-cell metal foam. The authors observed that there was a significant improvement of about 1.2% in the melting process when the phase change material was doped with nanoparticles. The combined effect of bio-based coconut oil and CuO nanoparticles in solar thermal applications using experimental and theoretical approaches was studied by Al-Jethelah et al. [15]. They concluded that adding nanoparticles to PCM improved the melting process significantly. Regarding the heating and cooling processes of PCMs, experimental studies on the influence of Al_2_O_3_ with multi-walled-carbon nanotubes (MWCNTs) were undertaken by Aqib et al. [5]. The results revealed that the paraffin wax composite having 6 wt % of MWCNTs was better than a sample with PCM only. Arici et al. [16] numerically investigated the impact of adding internal fins and nanoparticles on the PCM melting rate. According to the study, fins and nanoparticles increased the melting rate by around 50%. The influence of CuO nanoparticles in paraffin wax beneath the V-corrugated solar still was experimentally analyzed by Behura and Gupta [17]. They came to the conclusion that the daily yield of a 0.3% nanoparticle was 2.04 L/m^2^.

Rufuss et al. [18] used graphene oxide, titanium oxide, and copper oxide nanoparticles in paraffin wax to enhance the thermal performance of solar still. Adding GO, TiO_2_, and CuO nanoparticles to the paraffin wax enhanced the thermal conductivity by 101% and 29%, respectively. Furthermore, it was observed that using metal oxide-based nanoparticles in the paraffin wax improved the thermal performance of solar still by 35% and 26% using CuO and TiO_2_ nanoparticles, respectively, compared to the conventional solar still. However, there is a decrease of about 7.6% using graphene oxide-based nanoparticles in paraffin wax compared to the conventional solar still.

Carbon-based nanoparticles are attracting greater interest for energy storage applications. He et al. [19] enhanced the thermophysical properties of PCM doped using carbon-based nanoparticles and evaluated the thermal performance. It was concluded that the carbon-based nanoparticles in the phase change material improved the chemical and thermal stability, significantly reducing the phase change transition time. It was also reported that the prepared sample could be well suited for energy storage, especially in solar thermal systems. The effect of using nano-based PCM with 15 wt % instead of a conventional PCM was investigated experimentally by Kandeal et al. [20]. As a result of nano-based phase change material in the basin of the solar still, the daily yield was enhanced by about 113% compared to the traditional solar still. Liu et al. [21] used Al/C hybrid nanoparticles with a Na_2_SO_4_⋅10 H_2_O PCMs system to boost the thermal conductivity. The thermal characteristics results showed a significant improvement in the thermal conductivity of about 26.41% compared to the PCM without a nanoadditive. In a theoretical study, Mahdi et al. [22] employed a 5% volume fraction of nanoparticles in PCM to boost heat transfer in a shell and tube energy storage system. For this purpose, the solidification time was reduced to 94% compared to the single PCM module. To improve the productivity of solar stills, Kumar et al. [23] used PCM and nano PCM. In comparison to traditional productivity, they found that previous modifications increased productivity by 51.22% and 67.07%, respectively. Parsa et al. [24,25] used gold (Au), silver (Ag), and titanium oxide (TiO_2_) as metal oxide nanoadditives in the fluid and experimentally evaluated the thermal performance of a double-slope solar still. They found that using a Ag-based solar still increased efficiency by 38.2% compared to the conventional system. Shanmugan et al. [26] used nano-doped PCM in the bottom of the basin to enhance the rate of fresh water produced from the conventional solar still. It was reported that the use of nanoparticles in the PCM enhanced the rate of fresh water up to 7.46 L/m^2^ compared to the yield of fresh water obtained from the CSS. The influence of copper oxide and aluminum oxide nanoparticles in phase change material for enhancing the fresh water from CSS was experimentally analyzed by Shoeibi et al. [27]. It was reported that an optimized volume concentration of 0.3% by weight of CuO and Al_2_O_3_ nanoparticles in the phase change material enhanced the rate of fresh water produced by 55.8% and 49.5%, respectively, with a CuO nano-coated absorber.

In an experimental study, Vigneswaran et al. [28] investigated the effect of using two phase change materials in the basin of a solar still. They concluded a 19.56% daily yield enhancement compared to the reference still. The thermophysical characteristics of graphene nanoparticles dispersed erythritol PCM for thermal energy storage applications were investigated by Vivekananthan and Amirtham [29]. The addition of 1 wt % graphene increased thermal conductivity by 53.1% while lowering latent heat enthalpy by only 6.1%, according to the findings. Rufuss et al. [30] used nanoparticle-enhanced phase change material (NPCM) as heat storage to improve the productivity of a solar still. Compared to a traditional solar still, the experimental results showed a 35% increase in productivity. Yang et al. [31] concluded that all studies increased the phase change rate due to the addition of nanoparticles. The dispersion of nanoparticles inside the PCM improved the system performance studied by Zhou et al. [32]. They concluded that heat flux increased by about 2.9% as the amplitude of the wavy wall increased.

According to the literature review, adding nanoparticles to paraffin wax in hemispherical solar distillates has not been studied. The aim of this study was to evaluate the yield improvements in a hemispherical solar distillation device containing PCM via Al_2_O_3_ nanoparticles. There is a delay in heat acquisition and storage when using PCM due to its low thermal conductivity (0.25 W/m K), so the use of PCM in improving distillation systems has remained largely underutilized. Commercial paraffin wax with a thermal conductivity of 0.25 W/m K is used as an energy storage medium. On the other hand, Al_2_O_3_ nanoparticles are characterized as having high thermal conductivity (38.5 W/m. K) and are low-cost. Therefore, nanoparticles have been incorporated with phase change materials in heat storage applications to enhance the thermal conductivity of distillation systems.

## 2. Preparation of PCM with Nanoparticle

**Figure 1:**
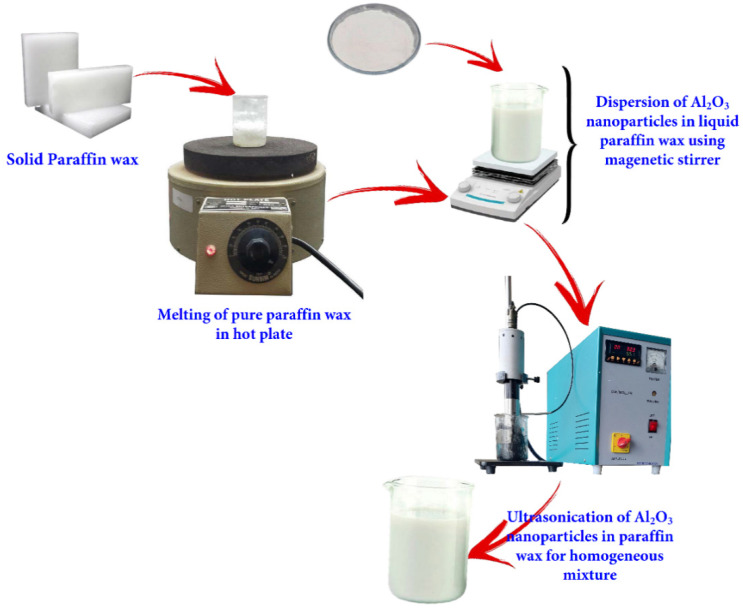
Preparation of paraffin wax with Al_2_O_3_ nanoadditive.

Initially, the weight of the paraffin wax (750 g) was measured using an electronic scale, followed by measuring the weight of the nanoparticles. Then, the paraffin wax was melted into liquid form in a hot plate gauge, and the measured Al_2_O_3_ nanoparticles (3 g) were dispersed. In the first stage, Al_2_O_3_ nanoparticles were mixed using a magnetic stirrer for 30 min, followed by a homogeneous mixture using an ultrasonicator by a probe sonicator for 45 min. The process of the preparation of nano-enhanced paraffin wax is shown in Figure 1. Table 1 gives the thermo-physical properties of the used PCM, and Table 2 shows the specifications of the Al_2_O_3_ nanoparticles. SEM analysis was used to measure the particle size and geometry of the nanoparticles procured from the supplier.

### Characterization of Nanoparticles

**Figure 2:**
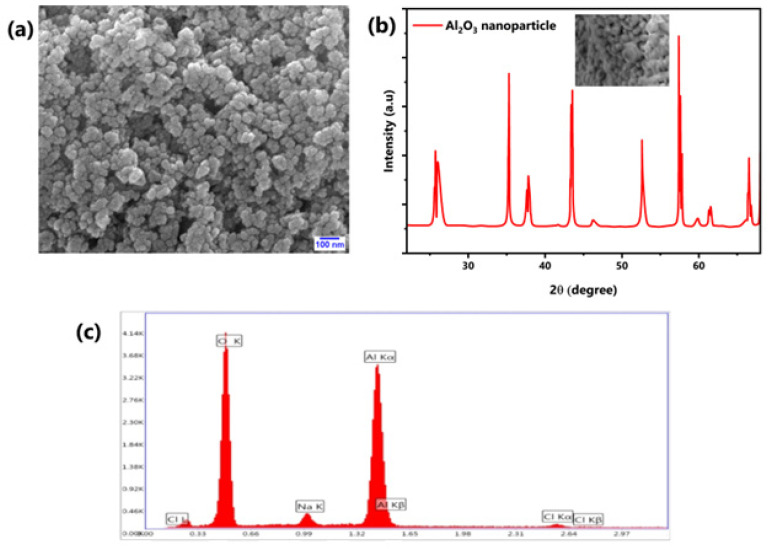
(**a**) SEM image of Al_2_O_3_ nanoparticles. (**b**) XRD pattern of Al_2_O_3_ nanoparticles. (**c**) EDS spectrum of the nanoparticles.

Figure 2 shows the various characterizations of the procured Al_2_O_3_ nanoparticles. SEM images of the Al_2_O_3_ nanoparticles revealed that the particles are spherical in shape and they are clustered. The average diameter of the Al_2_O_3_ nanoparticle is 13.6 nm. Similarly, the XRD patterns revealed that there are 7 large peaks with the existence of 3 small peaks. These clearly showed the crystalline structure of the obtained Al_2_O_3_ nanoparticles. On the other hand, EDS spectrum analysis of the purchased nanoparticles revealed the presence of α-Al with small traces of Na and Cl, which are negligible. Additionally, it was found that there is a greater presence of O along with α-Al.

**Figure 3:**
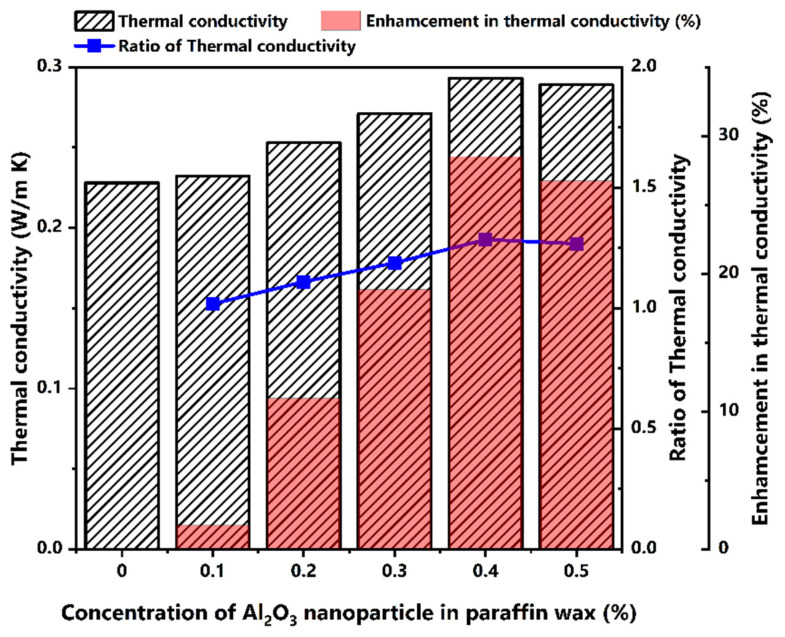
Variations in thermal conductivity, the ratio of thermal conductivity, and enhancement in thermal conductivity.

Stability issues of the nanoparticle in phase change material is another critical parameter in assessing the properties. In particular, zeta potential and thermal conductivity enhancement ratio are the critical parameters to be analyzed. The stability of the nano-dispersed paraffin wax at different compositions was analyzed using a zeta potential analyzer (Malvern Zetasizer, Malvern Panalytical, Malvern, U.K.). The zeta potential results of the as-prepared samples are tabulated in Table 3. Based on the results, it was found that the paraffin wax with 0.4% Al_2_O_3_ nanoparticles is more stable compared to the other samples. The paraffin wax with 0.4% Al_2_O_3_ nanoparticles showed excellent stability, whereas the particles with 0.3% and 0.5% Al_2_O_3_ nanoparticles showed good stability. Similarly, the nanoparticle concentrations of 0.1% and 0.2% showed incipient instability. A physical examination of the prepared samples revealed a small agglomeration of the nanoparticles at the bottom using 0.3% and 0.5% Al_2_O_3_ nanoparticles along with paraffin wax. The thermal conductivity levels of pure paraffin wax and paraffin wax with different compositions of Al_2_O_3_ nanoparticles compared with that of pure paraffin wax were measured using the TEMPOS thermal analyzer. The results of the thermal conductivity of paraffin wax and paraffin wax with 0.1%, 0.3%, 0.4%, and 0.5% Al_2_O_3_ nanoparticles are plotted in Figure 3, along with the thermal conductivity enhancement ratio. It can be seen that there is a significant enhancement in the thermal conductivity of paraffin wax with 0.4% Al_2_O_3_ nanoparticles. However, the thermal conductivity of the paraffin wax using 0.5% Al_2_O_3_ nanoparticles decreased.

## 3. Experimental Setup and Procedure

**Figure 4:**
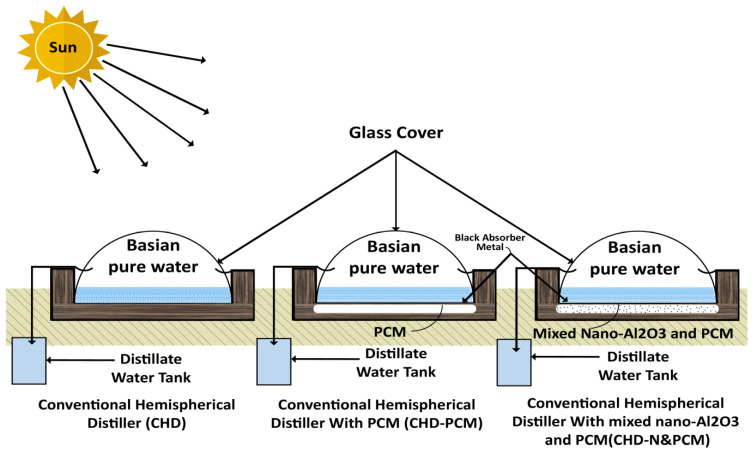
Graphical representation of the different configurations of hemispherical solar still.

The experimental test rig consists of three identically shaped hemispherical solar distillers. The distillers are made of circular-shaped steel plates with a diameter of 38 cm and a depth of 4 cm. The still basins are painted black to increase solar absorption, and the glass cover has a diameter of 40 cm and a thickness of 3 mm. The still basin is secured in a wooden box 25 cm thick filled with thermal insulation to insulate the inner sides. The first distiller is a CHD with a 1 cm high level in the distilled basin. The second distiller is filled with 1 cm thick paraffin wax (PCM) with a total mass of 0.75 kg, and it is also utilized in the bottom of the distiller and protected by a galvanized steel plate. The third distiller is filled with the same PCM with Al_2_O_3_ nanoparticles with a mass of 3 g. The condensed water flows down from the sides of the glassy cover and is collected through the collection channels. The employed distillation apparatus is depicted in Figure 4.

**Figure 5:**
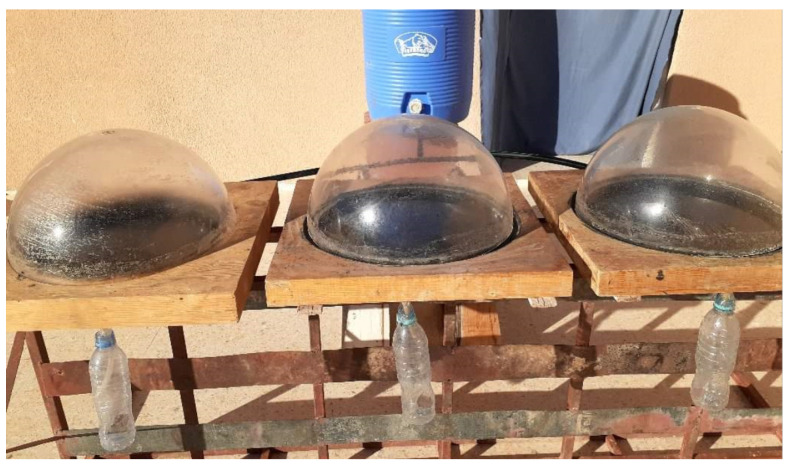
Experimental setup.

Figure 5 shows the photographic outlook of the experimental test rig. The first distiller is conventional, the second distiller has PCM at the bottom of the basin, and the third distiller is modified by mixing nano-Al_2_O_3_ with PCM at the basin plate reservoir. Experiments were carried out in the same weather circumstances, and their yields were compared with those of the conventional hemispherical distiller.

The temperature was measured using thermocouples (type K) that were placed throughout the distiller apparatus parts. Thermocouples are normally used to measure the temperature of different solar still elements. On the two faces of the acrylic cover, the thermocouple was placed to measure the cover temperature, and the average was taken. Similarly, the variations in the water temperature were measured on an hourly basis. In addition to the thermocouple placed for measuring the cover and water temperatures, the temperatures of PCM and PCM with the Al_2_O_3_ nanoadditive were also measured. Thermocouples are also employed to measure the ambient temperature. A solar power meter is normally used to measure the global solar radiation on the cover surface. The temperatures of all the elements of the hemispherical solar still with PCM, with nanoparticle-enhanced phase change material, and without any energy storage were recorded on a 24-h basis with 1 h time intervals. Similarly, the temperatures of PCM and PCM with Al_2_O_3_ nanoparticles estimated the amount of energy stored for the charge and discharge of heat distributed to the water placed in the basin. The volume of water collected in the distillate chamber was measured using a cylindrical graduated flask connected to the distillate collector using a flexible hose. For the outdoor conditions of El-Oued, Algeria, the experiments were conducted for HSS with PCM, PCM with Al_2_O_3_ nanoparticle, and HSS without energy storage. The uncertainties and errors of the instruments used in the experimental measurements were investigated. Similarly, the ambient parameters were measured using a power meter and anemometer, and the individual uncertainties are calculated using Equation (1).

(1) $$U\left(y\right)=\sqrt{{\left(\frac{𝜕Y}{𝜕x_{1}}U_{1}\right)}^{2}+{\left(\frac{𝜕Y}{𝜕x_{2}}U_{2}\right)}^{2}+\ldots {\left(\frac{𝜕Y}{𝜕x_{n}}U_{n}\right)}^{2}}$$

(2) $$U_{me,w}=\sqrt{{\left(\frac{𝜕m}{𝜕n_{1}}U_{me,w}\right)}^{2}}$$

Using Equation (2), the uncertainty in measuring potable water produced is calculated; using Equation (3), the total uncertainty that occurred in the daily thermal efficiency is estimated.

(3) $$U_{ƞ}=\sqrt{{\left(\frac{𝜕ƞ}{𝜕m}U_{me,w}\right)}^{2}+{\left(\frac{𝜕ƞ}{𝜕I\left(t\right)}U_{I\left(t\right)}\right)}^{2}}$$

From Equations (2) and (3), the uncertainty that occurred in measuring and calculating potable water and daily thermal efficiency are ±3.6% and ±2.4%, respectively. The values of the uncertainty that were obtained from the measuring devices are tabulated in Table 4.

## 4. Results and Discussion

**Figure 6:**
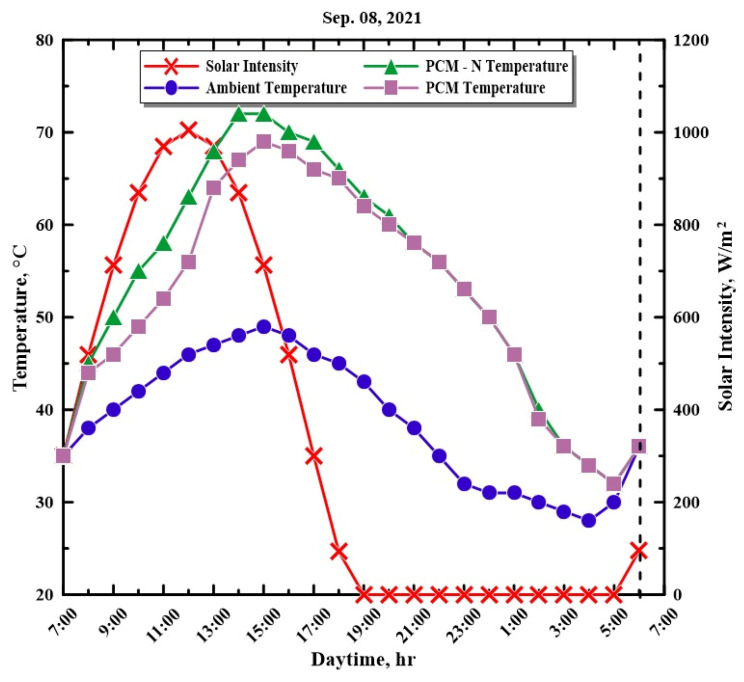
Variations in the temperature of the water, PCM, NPCM, ambient temperature, and solar radiation during the experiment.

In this experiment, three hemispherical distillers were developed and operated, which took place on 8 September 2021 in southeastern Algeria (6°47′ E and 33°30′ N). The data were collected for twenty-four hours, from 7:00 a.m. to 6:00 a.m. the next day. The performance of solar distillers is greatly influenced by sun intensity and ambient temperature. Thus, it was necessary to measure and record the required data every hour throughout the experimental day. Figure 6 depicts the hourly variation in the solar intensity, ambient temperatures, PCM, and PCM-N temperatures during the trial hours. The solar intensity increased until it reached its peak of 1004 W/m^2^ at 12:00 p.m. Then, as time passed, after reaching the maximum solar intensity, it gradually decreased until it approached zero after sunset. At the same time, the ambient air temperature varied between 28 °C and the highest recorded ambient temperature of 49 °C at 3:00 p.m. We also found that the PCM temperature ranged between 32 and 69 °C, and the PCM-N temperature ranged between 32 and 72 °C, which might be due to the enhancement in the thermal conductivity by the addition of nanoparticles, which leads to heat diffusion through the PCM.

**Figure 7:**
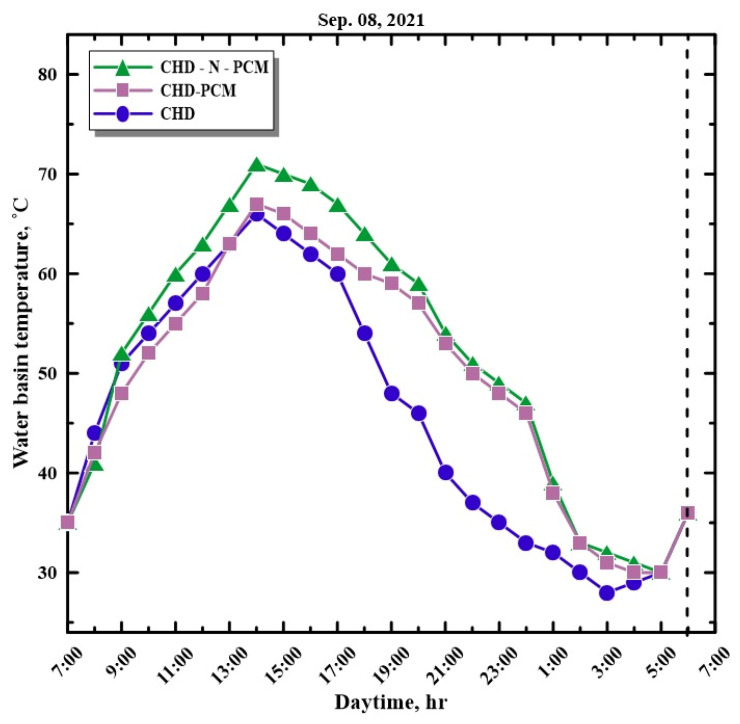
Hourly variation in water basin temperature with time.

Figure 7 shows the variations in the temperature of water recorded from the SS using different configurations from 7:00 a.m. to 6:00 a.m. on 8 September 2021. From the experimental results, the HSS using PCM with Al_2_O_3_ nanoparticles was higher than the HSS with PCM as energy storage and the HSS without PCM during the peak solar radiation condition. This may be due to the enhanced thermal conductivity of paraffin wax with metal oxide nanoparticles. Additionally, it was observed that the water temperature of the HSS without paraffin wax in the basin was higher during the sunshine hours, as the paraffin wax absorbs the heat from the water, which simultaneously reduces the temperature. The melting temperature of wax improves the storage of energy, which is used during the night hours for enhanced water temperature for a higher rate of evaporation. The peak water temperature recorded at 14:00 h (2:00 p.m.) from the HSS using PCM with the Al_2_O_3_ nanoadditive was 71 °C, whereas the peak water temperatures of the HSS with paraffin wax as energy storage and without energy storage were 67 and 66 °C, respectively.

**Figure 8:**
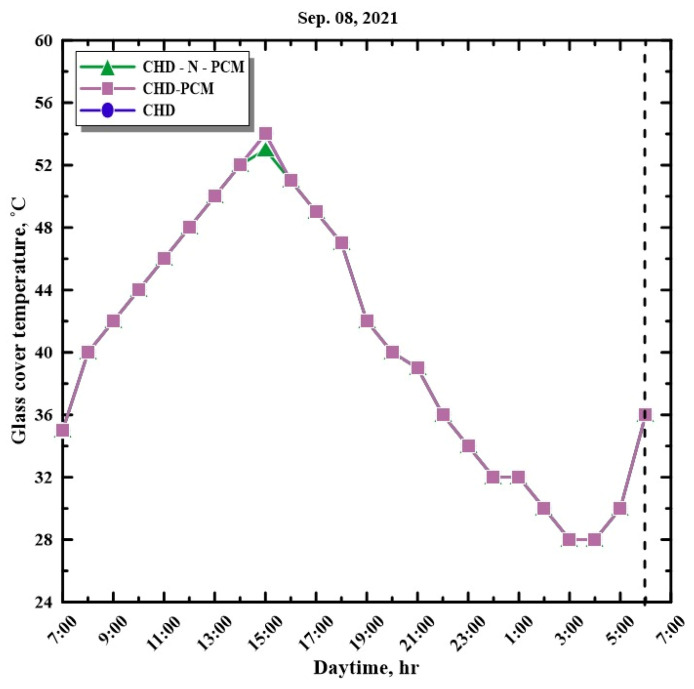
Hourly variation in glass cover temperature with time.

**Figure 9:**
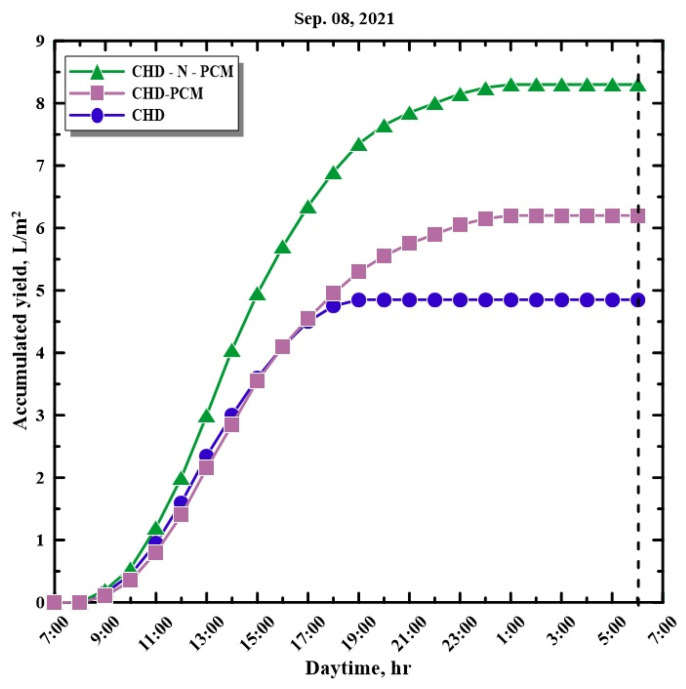
Hourly variation in accumulated yield with time.

Figure 8 shows the variations in the temperature of the acrylic cover on the external surface and recorded from the SS using different configurations from 7:00 a.m. to 6:00 a.m. on 8 September 2021. The external cover temperature variations show that the temperatures of the covers from HSS using nano-enhanced paraffin wax, HSS with paraffin wax, and HSS without energy storage were almost identical. It is observed that the temperature of the hemispherical cover surface reached a maximum of 54 °C for HSS with paraffin wax in the basin and conventional HSS, whereas the cover temperature of HSS with nano-enhanced paraffin wax was 53 °C. According to the obtained results for the temperatures of both the glass cover and the PCM with and without nanoparticles, the maximum temperature difference was found with CHD-N-PCM, which is the main driving force for evaporation and condensation (double-diffusive). Consequently, the addition of nanoparticles enhances productivity. Figure 9 shows the variations in the accumulated yield from the SS with a hemispherical cover and using various configurations from 7:00 a.m. to 6:00 a.m. on 8 September 2021. It is observed that the cumulative yield of potable water produced from the hemispherical solar still using nano-enhanced paraffin wax is higher than the HSS with paraffin wax and the HSS without any energy storage medium. The daily accumulated yields for CHD, CHD-PCM, and CHD-N-PCM are 4.85, 6.2, and 8.2 L/m^2^, respectively. Table 5 shows that the daily yield productions from CHD, CHD-PCM, and CHD-N-PCM are 4.85, 6.20, and 8.30 L/m^2^/day, respectively, achieving improvement percentages of 27.84% and 71.13% compared to the conventional hemispherical distiller.

The thermal performance of any solar still completely depends on the cumulative yield obtained. The daily thermal efficiency of the hemispherical cover solar still is the product of cumulative yield and latent heat to the input solar radiation with the associated basin area. Mathematically, it is given as,

(4) $$\eta_{daily\;thermal\;efficiency}=\frac{\sum m_{e}\times h_{fg}}{I\left(t\right)\times A_{w}\times 3600}\;$$

where *m* is the amount of fresh water collected (kg/h), *h_fg_* is the latent heat of vaporization (kJ/kg), *I*(*t*) is the incident global radiation falling on the cover surface (W/m^2^), and A is the area of solar still (m^2^).

The latent heat of vaporization with respect to different water temperatures is estimated using Equation (5), and it is mathematically expressed as,

(5) $$h_{fg}=2.4935\times 10^{6}\left[1-9.4779\times 10^{-4}T_{w}+1.3132\times 10^{-7}T_{w}^{2}-4.794\times 10^{-9}T_{w}^{3}\right]$$

It is seen that the daily efficiency of CHD-N-PCM is much better than CHD-PCM and CHD. The average amount daily efficiencies of CHD, CHD-PCM, and CHD-N-PCM are 40.66%, 51.79%, and 69.18%, respectively.

## 5. Comparative Analysis of Fresh Water Produced from Different Solar Stills in Previous Literature and the Present Study

A comparison of the current research with previously published publications that are comparable is given in Table 6. The results show that the accumulated yield of a hemispherical solar distiller with paraffin wax (CHD-PCM) increased by 27.84% compared to CHD, and the cumulative yield increased by 71.13% when using Al_2_O_3_ nanoparticles dispersed in paraffin wax (CHD-N-PCM). The enhancement of fresh water production from the HSS unit with NPCM beneath the basin is largely due to the higher thermal energy storage ability of the metal oxide nanoparticles in the paraffin wax for better thermophysical properties. Moreover, Table 4 shows that using cascade SS with Al_2_O_3_ [35] achieves a minimum yield enhancement equal to 22%. However, SS with Al_2_O_3_ via a running fan attains the maximum yield enhancement of 125% (Kabeel et al. [36]).

## 6. Economic Evaluation

Estimating the payback period is important in the economic study of solar distillers. The daily accumulated yields of CHD-N-PCM, CHD-PCM, and CHD were recorded on 8 September 2021 for 24 h. Table 7 shows the results of a detailed economic analysis to establish the time required to recoup the total cost of CHD, CHD-PCM, and CHD-N-PCM. The payback periods for CHD, CHD-PCM, and CHD-N-PCM are 31, 25, and 19 days to recover the whole cost.

## 7. Conclusions

The present study deals with the experimental investigation of hemispherical solar stills loaded with paraffin wax and Al_2_O_3_ nanoparticle-doped paraffin wax (composite PCM) for improving fresh water production. In this method, paraffin wax is loaded at the bottom of the basin with a thickness of 10 mm of hemispherical solar still. Furthermore, the Al_2_O_3_ nanoparticles are doped in paraffin wax to improve the fresh water yield. This approach is not only efficient but also easy to implement. It also has no effect on the surrounding environment. This recently proposed approach offers considerable advancements as compared to prior research and their respective solutions. The following is a list of the conclusions reached:

- The daily distillate production from CHD is equal to 4.85 L/m^2^. However, it is equal to 6.2 L/m^2^ from the distiller CHD-PCM and 8.3 L/m^2^ from the distiller CHD-N-PCM.
- The average daily efficiencies of CHD, CHD-PCM, and CHD-N-PCM are 40.66%, 51.79%, and 69.18%, respectively.
- The addition of paraffin wax increases the daily yield and efficiency of a hemispherical distiller to 27.84% and 27.38%, respectively, compared to the CHD.
- Adding nanoparticles of Al_2_O_3_ to paraffin wax enhances the daily yield and efficiency of a hemispherical solar distiller with 71.13% and 70.16%, respectively, compared to the CHD.
- Compared to the distiller CHD-PCM, improved yield and efficiency in the distiller CHD-N-PCM are achieved, with higher rates of 33.87% and 33.58%, respectively.
- The payback period required to recover a conventional hemispherical solar distiller is 31 days. This period is equal to 25 days for a hemispherical distiller using paraffin wax (CHD-PCM). However, a hemispherical distiller using paraffin wax via Al_2_O_3_ nanoparticles (CHD-N-PCM) is even less, 19 days.
- Increasing the fresh water production and efficiency of the hemispherical solar distiller may be accomplished by the use of paraffin wax that has been modified with Al_2_O_3_ nanoparticles. Therefore, using paraffin wax with Al_2_O_3_ nanoparticles is recommended to be considered in such applications. However, the use of metallic oxide nanoparticles with paraffin wax is limited to less than 0.5%, as the increase in volume concentration leads to particle agglomeration and sedimentation. It is also seen that the increase in the volume concentration of nanoparticles with paraffin wax leads to a reduction in the thermophysical property (thermal conductivity).

### Future Recommendations

From the analysis, the use of low-cost nanoparticles, especially adding carbon-based materials, can be used as an additive to paraffin wax for improved thermal conductivity and thermophysical properties.

## Figures and Tables

**Table 1:** PCM properties (thermophysical).

Property		Melting Temperature (°C)	Latent Heat (kJ/kg)	Thermal Conductivity (W/m K)	Density (kg/m^3^)	Specific Heat Capacity (kJ/kg K)
Value	Solid	56	226	0.228	886	2.17
	Liquid			0.251	753	3.06

**Table 2:** Specifications of Al_2_O_3_ nanoparticles.

Property	Thermal Conductivity (W/m K)	Density (g/cm^3^)	Specific Heat Capacity (J/kg K)	Color (Appearance)	Average Particle Size (nm)	Morphology
Present study	38.5	3.89	880	White	13.6 nm	Spherical
Chandrasekar et al. [33]	-	3.88	729	White	43 nm	Spherical
Ali et al. [34]	29–38	4.43	-	White	30–60 nm	Spherical

**Table 3:** Zeta potential analysis of paraffin wax with different compositions of Al_2_O_3_ nanoparticles.

Composition of Nanoparticle in Paraffin Wax	Zeta Potential (ς/mV)	Behavior
0.1	−15	Incipient instability
0.2	−32	Incipient instability
0.3	−45	Good stability
0.4	−62	Excellent stability
0.5	−42	Good stability

**Table 4:** Uncertainty, range, and accuracy of the measuring instruments.

Instrument	Range	Accuracy	Uncertainty
Solar power meter	0–3500 W/m^2^	±10 W/m^2^	3.1%
Thermocouple	−150–600 °C	±0.1 °C	1.2%
Graduated cylinder	0–500 mL	±1 mL	3.6%

**Table 5:** Cumulative distillation output of CHD, CHD-PCM, and CHD-N-PCM during trial hours.

Solar Still	Day Time Production (L/m^2^)	Overnight Fresh Water Yield (L/m^2^)	Cumulative Yield (L/m^2^)	Enhancement (%)
CHD	4.85	0	4.85	-
CHD-PCM	5.30	0.90	6.20	27.84
CHD-N-PCM	7.35	0.95	8.30	71.13

**Table 6:** Comparison between the daily yield enhancement of the current work and previously published work.

Literature	Country	Solar Still Type	Medium of Enhancement	Improvement in Fresh Water Yield (%)
Parsa et al. [24]	Iran	Single slope	- Ag	26.3
Rashidi et al. [35]	Iran	Cascade	- Al_2_O_3_	22
Kabeel et al. [36]	Egypt	Single slope	- Al_2_O_3_ with outside heat exchanger	116
Kabeel et al. [37]	Egypt	Tubular	- PCM	115.0
Chaichan and Kazem [38]	Iraq	Single slope	- PCM - Combination of PCM with a nano-Al_2_O_3_	10.38 60.53
Kabeel et al. [39]	Egypt	Single slope	- Al_2_O_3_ - Al_2_O_3_ with running fan	89 125
Kabeel et al. [40]	Egypt	Pyramid shaped	- Graphite as absorber plate with cover cooling	107.7
Kabeel et al. [41]	Egypt	Single slope	- Absorber plate coated with CuO nanoparticles	25.3
Present work	Algeria	Hemispherical	- PCM - Mixed nano-Al_2_O_3_ with PCM	27.84 71.13

**Table 7:** Fabrication cost of CHD, CHD-PCM, and CHD-N-PCM.

	CHD	CHD-PCM	CHD-N-PCM
Manufacturing cost (USD)	68	68	68
Al_2_O_3_ nanoparticle price (USD)	-	-	0.5
PCM price (USD)	-	1.5	1.5
Maintenance cost (USD)	0.5	0.5	0.5
Total cost (USD)	68	69	70
Potable water produced (L/m^2^/day)	4.85	6.2	8.3
CPL of potable water produced (USD)	0.5	0.5	0.5
The cost of daily water production (USD)	2.2	2.8	3.75
Payback period (days)	31	25	19

## Data Availability

The data will be made available on personal request to the authors.

## Abbreviations

PCM	Phase change materials
SS	Solar still
CHD	Conventional hemispherical distiller
CHD-PCM	Conventional hemispherical distiller with PCM
CHD-N-PCM	Conventional hemispherical distiller with nano-Al_2_O_3_ and PCM
